# The Visions of Mary Czajka1Read at the annual meeting of Medical Society of the State of New York, January 30, 1907.

**Published:** 1907-03

**Authors:** F. E. Fronczak

**Affiliations:** Buffalo, N. Y.; 806 Fillmore Avenue


### The Visions of Mary Czajka.[1]

By F. E. FRONCZAK, A. M., M. D., Buffalo, N. Y.

ON May 23 1906, the inhabitants of Buffalo were startled by the news that a great prophetess and seeress was among them—one who was able not only to foretell the future, explain the past, but also to be in communion with the saints and God himself. On the afternoon of that day, a rumor was spread through East Buffalo that on the following day, a young girl would communicate with the Blessed Virgin and the saints and tell the people at large what are the wishes of heaven in regard to certain things to be done. On the following morning, promptly at 10 o'clock, as foretold, I received a message that a young girl was stiffened in a room and was talking with saints. I was asked to come in my professional capacity to examine her. I hurried there and found the street in front of the house, crowded with people. I could not get into the house as it was packed. Finally, after much struggle, I obtained entrance and there I saw, sitting in a chair, quite a handsome young Polish girl, aged 17 years; her hands extended; her eyes closed; anemic in appearance.

I tried to speak to her, but at that time she began to speak softly, repeating what were supposed to be the words of the Blessed Virgin to her. I examined her. She was perfectly stiff. I used all the force I could, but could not bend her hands or elbows; reflexes none; pupils widely dilated; insensitive to touch. I pricked her with needles and applied electricity to various parts

1. Read at the annual meeting of Medical Society of the State of New York, January 30, 1907.

of her body. She smiled, saying softly and pleasantly that the devil who was torturing her would drop head if he did not stop. "The Blessed Virgin," she said, "told me that unless the doctor" (and she seemed to know who I was, though I could see that her eyes were closed and insensitive to light) "would turn as black as the stove" and then she kept on speaking the following words: "The Blessed Virgin says that great earthquakes are coming; that thousands of people will be killed; the Blessed Virgin says that the world is getting worse and worse; that God will punish the whole world with fire, war, earthquake and diseases; the Blessed Virgin says she will strike Dr. Fronczak dead unless he stops burning me."

At that moment, I was testing the reaction of her pupils with a lighted match. She murmured, sighed and came to; she looked around bewildered. The people in the rooms, who had been upon their knees, arose and began to kiss her hands, her dress, and her feet, and to call upon the saints,—looking upon me as upon the very devil, and here and there muttering words of condemnation upon my head. When she came to, I examined her thoroughly. Aside from her anemic appearance, she seemed to be perfectly well. She had menstruated regularly for the past two or three years. She read the bible, attended church formerly, "but stopped because the priests were bad."

She told me that at 2 o'clock that same day she would have another vision. I requested Drs. William C. Krauss and James W. Putnam, nervous disease specialists, to join me in examining the young girl. They did so, but could find nothing pathological about her. She went through the same form of stiffening, and repeating almost all her former sayings.

Somebody told us that her brother, who was a tall, large man, seemed to influence her. He was 6 ft., 3 in. in height and weighed about 250 lbs., with leonine head, bushy hair and piercing eye. While she was in this semicomatose state, we watched her brother. We noticed that his lips were moving and with the movements of his lips the girl spoke. We requested this man to leave the room. The moment he did so, the girl came to. We called him in and the girl fell in her former semicomatose state. We charged him with hypnotizing the girl, which charge he denied. However, we learned that he formerly took lessons in hypnotism. After consultation, we told the people that the girl was not a saint, but was simply suffering from exaggerated hysteria; was hypnotized and spoke only on the suggestion of her brother. Some of the people turned upon the three physicians present and abused us. Dr. Krauss then proposed that the girl submit to him and that he should hypnotize her. After proceeding for a minute with the movements of his hands on the girl's forehead and suggesting to her that she fall asleep, the people, at first, and then the girl herself refused to have any experiments made upon our seeress. We told the people then that the whole affair was a fake. Many believed us and left the house immediately.

We notified the police who dispersed the crowd from the house. The press took up this matter and after telling the astonished citizens of Buffalo about the visions of the prophetess also began to throw cold water upon the whole affair and in a few days it quieted down. The surprising fact of this was first, the influence of the big brother upon this innocent, simple minded girl; second, the ease with which the populace believes in certain so-called supernatural visions; third and most surprising; is the idea that many so-called intelligent and highly educated women from the uptown district of Buffalo came in carriages and gave money for a chapel to be erected on that spot, and believed, with all the simple mindedness of the ignorant people, that this was a supernatural gift.

At present, after the exposure of the whole affair and explanation that it was exaggerated hysteria,—hypnotism and suggestion,—the house has been almost deserted. Only occasionally, usually on Sundays, a few simple minded folk come to the girl's house, pay twenty-five cents admission and listen to the always same repeated talk of the poor hysterical girl. And thus another seeress was exposed by medical procedure.

806 Fillmore Avenue.

---

Uterine Cancer and Pregnancy.—Vaginal hysterectomy or Cesarean section is recommended by Scheib (*Prager medicinische Wochenschrift,* No. 39, 1906), who has used the method four times.

The womb should be removed as soon as the condition is discovered, not waiting until the child is viable. During pregnancy the operation of hysterectomy per vaginam is easier than at other times. In these cases there is always danger of infecting the peritoneum if the abdominal route is followed. It is probably easier to make a complete operation through the vagina than through the abdomen. The vaginal route is preferred by the author whenever it is possible.—*Therapeutic Gazette,* February, 1907.

---

The Doctor—No; I don't know of any treatment that will cure a case of "exaggerated ego."

The Professor—And you a doctor! I've seen many a cure effected by homeopathic treatment—give the man affected with the exaggerated ego a swollen eye.—*Chicago Tribune.*

